# Sensitive Colorimetric Hg^2+^ Detection via Amalgamation-Mediated Shape Transition of Gold Nanostars

**DOI:** 10.3389/fchem.2018.00566

**Published:** 2018-11-27

**Authors:** Dong Xu, Shufang Yu, Yueqin Yin, Suyan Wang, Qinlu Lin, Zhiqin Yuan

**Affiliations:** ^1^National Engineering Laboratory for Rice and By-Products Further Processing, College of Food Science and Engineering, Central South University of Forestry and Technology, Changsha, China; ^2^Hunan Key Laboratory of Processed Food for Special Medical Purpose, College of Food Science and Engineering, Central South University of Forestry and Technology, Changsha, China; ^3^State Key Laboratory of Chemical Resource Engineering, Beijing University of Chemical Technology, Beijing, China

**Keywords:** gold nanostar, amalgamation, Hg^2+^ quantification, morphological transformation, LSPR sensing

## Abstract

Reliable and sensitive methods to monitor mercury levels in real samples are highly important for environment protection and human health. Herein, a label-free colorimetric sensor for Hg^2+^ quantitation using gold nanostar (GNS) has been demonstrated, based on the formation of Au-Hg amalgamate that leads to shape-evolution of the GNS and changes in its absorbance. Addition of ascorbic acid (AA) to GNS solution is important for quantitation of Hg^2+^, mainly because it can reduce Hg^2+^ to Hg to enhance amalgamation on the GNSs and stabilize GNSs. In addition to transmission electron microscopy images, the distribution of circular ratios of GNSs in the presence of 2 mM AA and various concentrations of Hg^2+^ are used to show the morphology changes of the GNSs. Upon increasing the concentration of Hg^2+^, the average circular ratio of GNSs decreases, proving GNS is approaching to sphere. The morphology change alters the longitudinal localized surface plasmonic resonance (LSPR) absorbance of the GNSs significantly. Under the optimum conditions, our sensor exhibits a dynamic response for Hg^2+^ in the range of 1–4,000 nM with a detection limit of 0.24 nM. Upon Increasing Hg^2+^ concentration, the solution color changes from greenish-blue, purple to red, which can be distinguished by the naked eye when the Hg^2+^ concentration is higher than 250 nM. Owing to having a high surface-to-volume ratio and affinity toward Hg^0^, the GNS is sensitive and selective (at least 50-fold over tested metal ions like Pb^2+^) toward Hg^2+^ in the presence of AA. Practicality of this assay has been validated by the analysis of water samples without conducting tedious sample pretreatment.

## Introduction

Mercury has been recognized as one of the most toxic elements. Once taken in by human body, it cannot easily be exerted and accumulates in organs, potentially resulting in serious diseases such as memory loss, kidney damage, and even death at its high content (Boening, 2000; Tchounwou et al., 2003; Baughman, 2006). Because mercury could be released from natural processes (Gustin, 2003; Leopold et al., 2010) and human activities (Streets et al., 2011, 2017; Driscoll et al., 2013) to the ecosystem, its contamination is still a rising global issue. Owing to its high toxicity, the safety contents for mercury in food and environment were set to be extremely low. For example, the maximum allowable levels of mercuric ion (Hg^2+^) in drinking water are as low as 10 and 30 nM according to the US Environmental Protection Agency (EPA) and the World Health Organization (WHO), respectively. Currently, common methods used for quantitation of Hg^2+^ include atomic absorption spectrometry (Hatch and Ott, 1968), inductively coupled plasma-mass spectrometry (Wu et al., 2011), and selective cold vapor atomic fluorescence spectrometry (Bendl et al., 2006). These methods involve commercial instruments and are rather complicated, time-consuming, costly, and inappropriate for on-site applications. In the last decade, colorimetric approaches (Du et al., 2013; Chansuvarn et al., 2015; Chen et al., 2015; Xu et al., 2015; Ding et al., 2016) using gold nanomaterials have become an alternative for Hg^2+^ quantitation due to their characteristics of simplicity, rapidness, selectivity, and sensitivity. In addition, the color change can be observed by the naked eye. Generally, these assays are based on the assembly or disassembly of gold nanoparticles having surface DNA molecules (Lee et al., 2007; Li et al., 2008; Xue et al., 2008), peptides (Si et al., 2007; Du et al., 2011) and organic molecules (Chai et al., 2010; Wang et al., 2018) through their high affinity for Hg^2+^. However, they are disadvantageous for the quantitation of Hg^2+^ in most real samples. To begin with, the limits of detection (LOD) of the most reported Hg^2+^ sensors are higher than 10 nM, limiting their real applications. Secondly, they sometimes do not provide satisfactory selectivity toward Hg^2+^ over some potential heavy metal ions such as Cu^2+^ and Cd^2+^ (Guo et al., 2011; Mehta and Kailasa, 2014; Wang et al., 2018), especially those based on coordination chemistry. Thirdly, stability of the functional gold nanoparticles in high-ionic-strength samples is a concern. Lastly, their functionalization usually involves complex procedures and time consuming. Therefore, developing label-free assays for accurate, selective and reliable mercury quantitation is still a challenging task.

Amalgamation—the reaction of mercury with other metals—is a normal but unique phenomenon in chemistry since mercury has a lower cohesive energy (0.69 eV) than other metals such as Au (3.7 eV) and Ag (2.95 eV). Amalgamation has become interesting in the field of nanotechnology. For example, the formation mechanism of Ag@Hg nanoalloys was investigated by monitoring *in situ* the growth of single silver nanoparticles with different shapes under a dark-field microscope (Liu and Huang, 2013). It has been confirmed that in the amalgamation process, mercury atoms are adsorbed onto the nanoparticle surfaces first, and then diffuse to interact with the silver atoms to form sphere nanoalloys. The process of slow diffusion of mercury into nanoparticles lattice was also evident in the formation of Au@Hg nanoalloys through transmission electron microscopy and high-angle annular dark field scanning transmission electron microscopy measurements, which was further supported with computer simulations (Mertens et al., 2011). The dynamic processes of amalgamation between gold nanorods and mercury atoms were investigated by conducting single nanoparticle spectroscopy and spectroelectrochemistry, revealing amalgam formation and consequent shape transformation from nanorods to spherical particles (Schopf et al., 2016). All of the results suggest that amalgamation can lead to shape-evolution of nanoparticle and nanoalloy formation, altering their localized surface plasmonic resonance. Accordingly, several selective assays toward Hg^2+^ ion detection using gold nanoparticles (Qi et al., 2012; Chaudhary et al., 2015; Park et al., 2016) and gold nanorods (Jin and Han, 2014; Schopf et al., 2017) have been developed based on nanoalloy formation. But, the sensitivity is not satisfied for real sample testings.

Gold nanostar (GNS) possesses unique physical and chemical properties originating from its branched nanostructures (Hao et al., 2004; Aad et al., 2011), which has become one of interesting gold nanomaterials and has been used in various fields such as surface-enhanced Raman scattering (Hrelescu et al., 2009; Esenturk and Walker, 2010), catalysis (Miao et al., 2011; Zhou et al., 2011; Yang et al., 2018) and biomedicine (Rodrĩguez-Oliveros and Sánchez-Gil, 2012; Wang et al., 2015). There are two plasmonic resonance modes for GNS–the longitudinal localized surface plasmonic resonance (LSPR) originating from its branches and the transverse localized surface plasmonic resonance (TSPR) depending on its core size. GNS with high yield could be readily and repeatedly prepared by using 4-(2-hydroxyethyl)-1-piperazineethanesulfonic acid (HEPES) as reducing, stabilizing and shape-directing agents. The LSPR of GNS can be readily tuned from the visible to the near infrared region by changing its shape, number density and length of its branches through altering the dosage of reagents (Hao et al., 2007; Liu et al., 2014). Because of its irregular structure and large surface-to-volume ratio, GNS has unique optical properties such as strong light scattering and extinction (de Puig et al., 2015). The incident electromagnetic radiation of GNS is prone to penetration to its protruding tips, allowing more efficient heat generation, and thus it is more suitable for photothermal therapy than gold nanorod (Rodrĩguez-Oliveros and Sánchez-Gil, 2012). The distinctive morphology of GNS governs the arrangement of atoms on its surface to form different types of facets, creating higher surface energy and greater catalytic activity (Zhou et al., 2011). More importantly, the sharp edges and tips provide GNS with a greater sensitivity toward local changes in the dielectric environment (Dondapati et al., 2010; Jana et al., 2015) as well as a large electric field enhancement (Esenturk and Walker, 2010; Liu et al., 2014), which make GNS as a good candidate for optical sensing applications. For example, surface-enhanced Raman scattering (SERS) signal of 4-mercaptobenzoic acid on a single GNS was detected without the need of forming hotspot through aggregation (Hrelescu et al., 2009). Through Hg^2+^ induced the formation of DNA capped-GNS dimer, SERS allows quantitation of Hg^2+^ in the linear ranges of 0.002–1 ng/mL. Among various shapes of gold nanomaterials, GNS is one of the most sensitive materials to the environmental change. GNS provides a refractive index sensitivity of 703 nm/RIU while that is only 44 nm/RIU for sphere gold nanoparticle (Chen et al., 2008). Streptavidin at the concentration of as lows as 0.1 nM led to an LSPR shift of 2.3 nm when using GNS (Dondapati et al., 2010). Nevertheless, its application in the convenient colorimetric assay for quantitation of mercury has not been reported.

In the present study, we propose a label-free, sensitive and selective method for Hg^2+^ detection by using GNS as a probe. The sensing principle is based on the LSPR change of GNS through mercury induced transformation of its morphology. A new term, namely, circular ratio that is highly related to the shape evolution of GNS in the presence of Hg^2+^ was used to evaluate the morphology change of GNSs. Transmission electron microscopy (TEM) images of GNSs at different Hg^2+^ concentrations were recorded to support our results. Because GNS has a high surface-to-volume ratio and forms amalgamate with mercury, it allows efficient capture of mercury. Correspondingly, our strategy for Hg^2+^ detection through UV-Vis spectroscopy measurement is very sensitive and the solution color change can be observed easily by the naked eye. The prominent sensitivity and selectivity of our assays facilitate their applications in the analysis of real samples.

## Methods

### Materials, reagents, and instruments

Hydrogen tetrachloroaurate(III) trihydrate (HAuCl_4_·3H_2_O). Ascorbic acid (AA, 99%) and HEPES were brought from Sigma-Aldrich (Milwaukee, WI, USA). Barium chloride (BaCl_2_), calcium chloride (CaCl_2_), cobalt sulfate heptahydrate (CoSO_4_·7H_2_O), copper sulfate pentahydrate (CuSO_4_·5H_2_O), hydrochloric acid (HCl, 36%), lead nitrate (Pb(NO_3_)_2_), manganese sulfate (MnSO_4_), mercuric nitrate (Hg(NO_3_)_2_), nitric acid (HNO_3_), potassium chloride (KCl), silver nitrate (AgNO_3_), sodium chloride (NaCl), sodium hydroxide, Tween 20 and zinc acetate dihydrate (C_4_H_6_O_4_Zn·2H_2_O) were purchased from Sinophorarm Chemical Reagent Corporation (Shanghai, China). Cr standard solution (1,000 μg/mL, Cr(NO_3_)_3_, GSB 04-1723) was obtained from National Center of Analysis and Testing for Non-ferrous Metals and Electronic Materials (Beijing, China). UV–Vis absorption spectra were recorded on a Shimadzu UV-1800 Spectrometer (Tokyo, Japan). The photos of GNS solution was taken by using an Iphone6 smartphone. Ultrapure water with an electric resistance of 18.2 MΩ was supplied with a Milli-Q water purification system (Billerica, MA, USA). Morphology characterization of GNSs was performed using a transmission electron microscope (TEM; JEM 1230, JEOL, Tokyo, Japan). All glassware used in the experiments was washed with aqua regia (HCl:HNO_3_ = 3:1 by volume) and rinsed with ultrapure water.

### GNS synthesis

GNSs were prepared using a similar procedure according to the literature (Xie et al., 2007). The method was very convenient and only HAuCl_4_ and HEPES were used, where HEPES serves as both a reducing agent and a soft template for structure formation. Typically, 20 mL of 0.1 M HEPES aqueous solution at pH 7.4 was mixed with 79 mL of ultrapure water in 100-mL volumetric flask, 823 μL of 24.28 mM HAuCl_4_ was then added to the mixture drop by drop, and finally ultrapure water was added to the mark. Next, the solution was kept undisturbed at room temperature for 30 min. The solution color varied from light yellow to mauve to greenish-blue, indicating the formation of nanoparticles. Accordingly, GNSs with various LSPR bands were obtained by varying the concentration of HEPES buffer (Xie et al., 2007; Dam et al., 2012; Liu et al., 2014). Finally, 90 μl of Tween-20 was added to 100 mL GNS solutions to stabilize the GNS and to reduce their adsorption onto the container surfaces, which were further stored at 4°C.

### Hg^2+^ detection

A stock solution of Hg(NO_3_)_2_ (1,000 g/L) was prepared for preparation of standard solutions through a serial dilution. To optimize the sensitivity and selectivity of our assay for Hg^2+^ detection, the solution pH value, incubation time, the LSPR of GNS and the AA content were optimized preliminarily. For the detection of Hg^2+^, 1.568 mL GNS solution (pH 5.0) and 32 μL of 0.1 M AA were added into a 2-mL centrifuge tube. After gentle shaking, aliquots (320 μL) of Hg^2+^ solution (ultimate concentrations: 0, 1, 5, 10, 50, 100, 250, 500, 1,000, 2,000, and 4,000 nM) were injected separately into each tube. After mixing and placing at room temperature for 150 min, the photos and UV-Vis spectra of the solutions were taken. To investigate the selectivity of our assay toward Hg^2+^, the above procedure was repeated, but Hg^2+^ was replaced with 100 μM of other metal ions, including Cu^2+^, Na^+^, K^+^, Ca^2+^, Mn^2+^, Pb^2+^, Co^2+^, Zn^2+^, Ba^2+^, and Cr^3+^. All the experiments are repeated three times.

### Analysis of real samples

Tap and pond water samples were collected from the campus of Central South University of Forestry and Technology. River water was collected from Xiang River (Changsha, Hunan). Before the analyses, each of the water samples was filtered through a 0.2-μm membrane. The spiked water samples were made by the addition of the standard Hg^2+^ solution to certain concentrations. All the water samples were mixed separately with 1.568 mL GNS solution (pH 5.0) and 32 μL of 0.1 M AA for analysis.

## Results and discussion

### Response of GNS toward Hg^2+^ in the presence of AA

**Figure 1:**
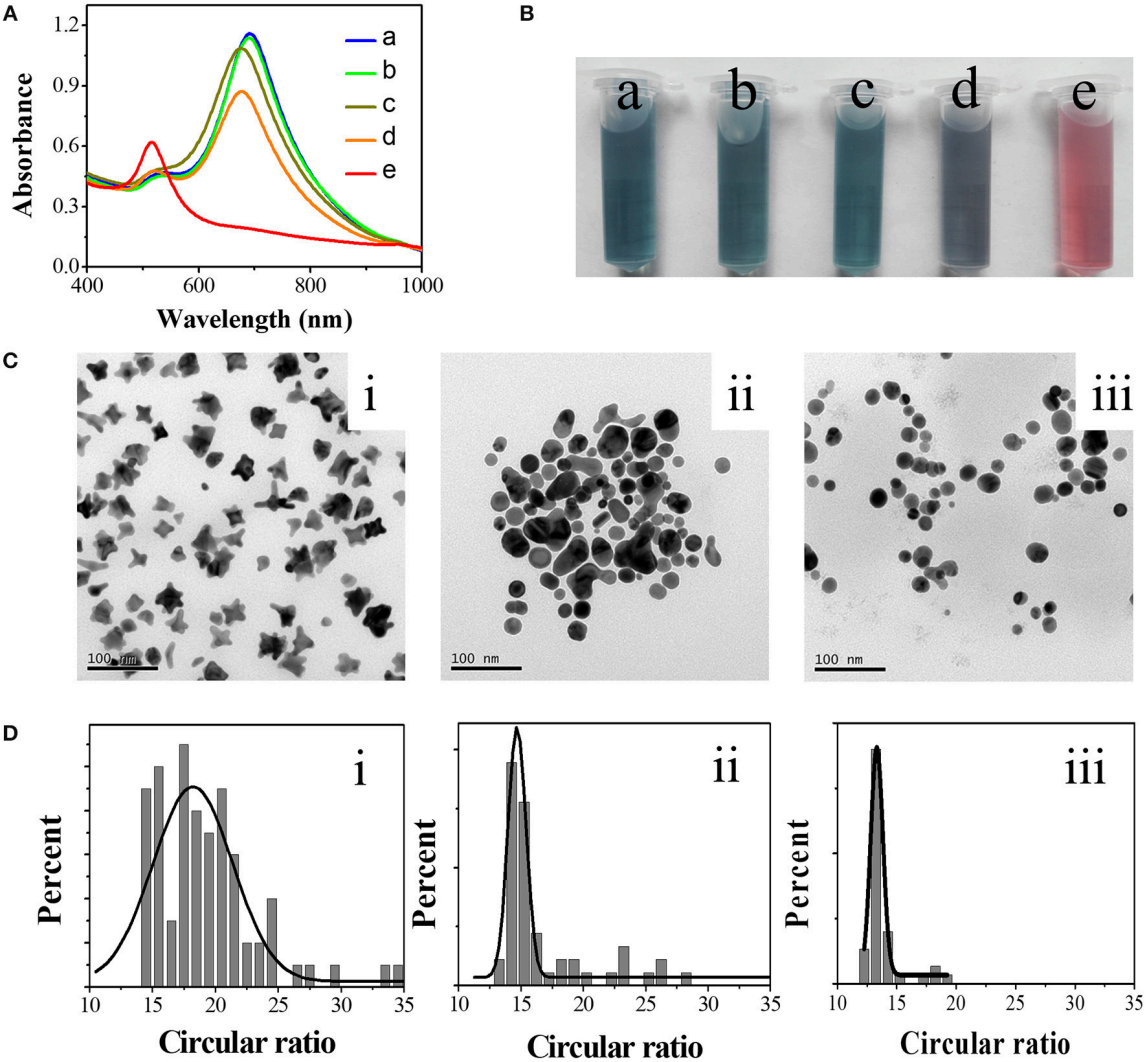
Response of GNS toward Hg^2+^ in the presence of AA. UV-Vis spectra **(A)** and photos **(B)** of solutions containing GNS (a), GNS + 2 mM AA (b), GNS + 4 μM Hg^2+^ (c), GNS + 2 mM AA + 4 μM Hg^2+^ (d), GNS + 2 mM AA + 8 μM Hg^2+^ (e). TEM images **(C)** and the statistical circular ratios **(D)** of the as-formed nanoparticles in the presence of 2 mM AA and 0 (i), 4 (ii), and 8 (iii) μM Hg^2+^, respectively. GNS was prepared in the presence of 75 mM HEPES.

**Figure 2:**
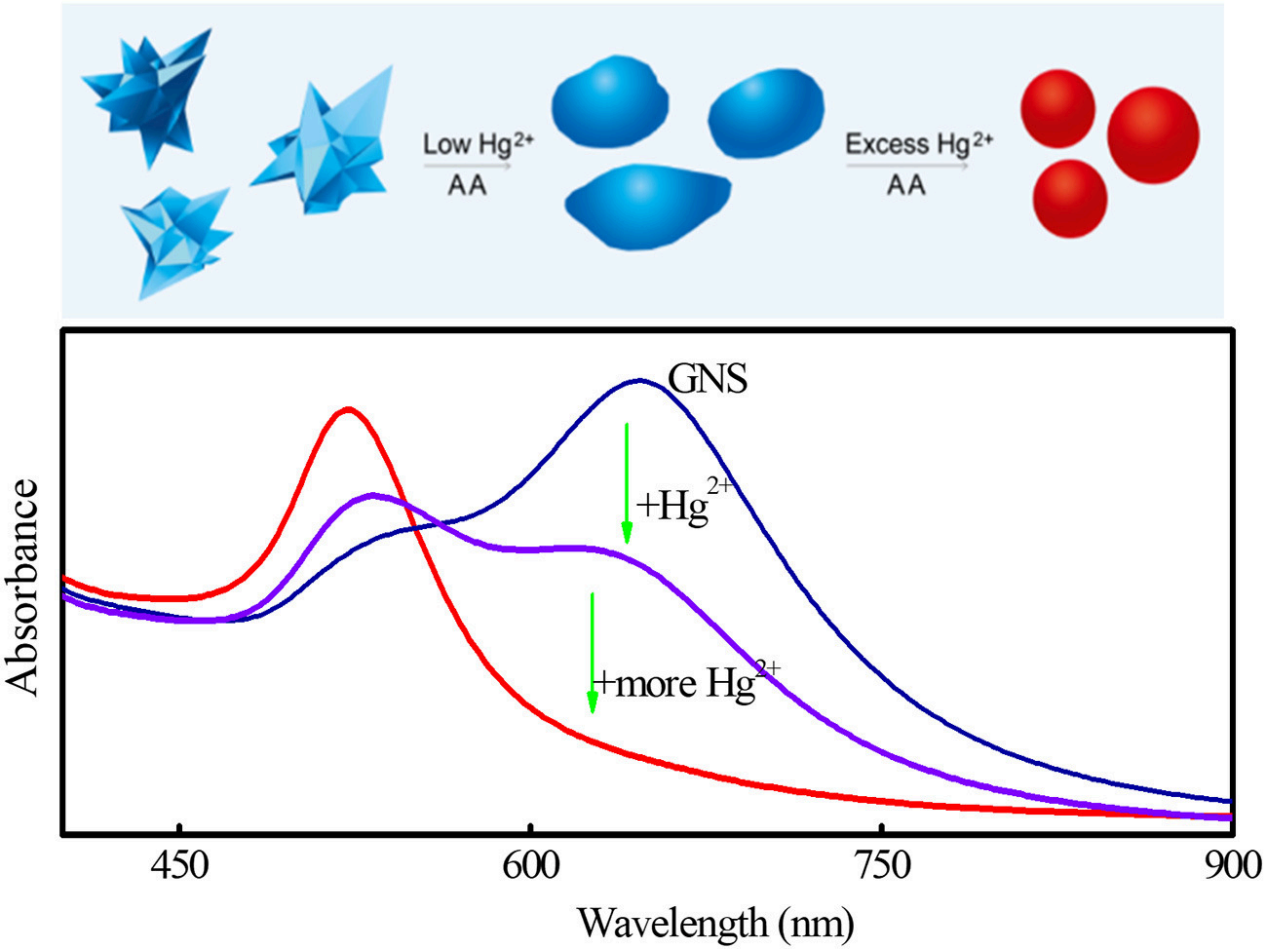
Scheme of sensing of Hg^2+^ through its inducing morphological transformation of GNS, leading to changes in the absorption spectra of GNS.

A typical GNS solution was prepared from HAuCl_4_ in the presence of 75 mM HEPES (pH 7.35), showing two absorption peaks at 520 and 690 nm that are assigned to the TSPR and LSPR bands of the GNS, respectively (spectrum a in Figure 1A). The solution color is greenish-blue as shown in photo (a) (Figure 1B). GNS was proven to have excellent response toward Hg^2+^ in the presence of AA (Figures 1A,B). The addition of AA is important because it allows reduction of Hg^2+^ to Hg^0^ that subsequently undergoes amalgamation with the GNS. The introduction of AA (final concentration 2 mM) to GNS solution did not induce any significant change in the UV-Vis absorption spectrum of GNS. With increasing the concentration of Hg^2+^, the absorption spectrum of GNS changes more significantly. In the presence of 2 mM AA and 4 μM Hg^2+^, the LSPR absorbance decreases from 1.15 to 1.08, accompanied with a blue shift from 690 to 672 nm. In contrast, in the absence of 2 mM AA, addition of 4 μM Hg^2+^ to GNS solution only caused a small decrease in the LSPR absorbance, with a blue shift of 10 nm in the LSPR. The blue shift is mainly because of adsorption of Hg^2+^ onto GNS surfaces that alters the surrounding dielectric constant (Ojea-Jiménez et al., 2012). When the concentration of Hg^2+^ is 8 μM in the presence of 2 mM AA, a single UV-Vis absorption peak at 520 nm was observed. Correspondingly, the solution color turned to red from greenish blue, indicating that the GNS transformed to form a spherical structure. The TEM images displayed in Figure 1C clearly show different morphologies of the nanoparticles under different conditions. The left TEM image supports the formation of GNSs, which possess various number of corners and tips. The GNSs with an average size of 28 nm contain 2–5 branches (Figure 1C). Besides GNSs, some spherical gold nanoparticles are existent in the presence of 4 μM Hg^2+^ as shown in the middle TEM image. Only spherical gold nanoparticles are existent in the presence of 8 μM Hg^2+^ as shown in the right TEM image. The TEM images support Hg inducing morphology changes in the GNSs, leading to changes in their absorption spectra. Similar changes of non-sphere nanomaterials to sphere nanoparticles have been reported (Rex et al., 2006; Schopf et al., 2017). We note that an excess amount of AA relate to Hg^2+^ in the solution is required for complete reduction of Hg^2+^ to Hg^0^, leading to greater shape evolution of GNS. For the purpose of evaluating shape transformation numerically, we introduced a new parameter—circular ratio (C). C can be calculated using Equation (1).

(1) $$\text{C}\;\text{=}\;{\text{P}}^{\text{2}}\text{/A}$$

where P is perimeter of each nanoparticle and A represents the area of the nanoparticle. Both P and A can be automatically acquired from the TEM images (Figure 1C) using the software ImageJ. In brief, after opening a TEM image and setting a threshold value to separate object and background, P and A of each GNS were calculated automatically. For a nanoparticle with a constant area, its P increases as its morphology becomes more irregular. In other words, the larger the C value is, the more irregular nanoparticles are. When the nanoparticles are perfect spheres, C is the smallest (4π) among all shapes. Thus, the shape transformation of GNSs can be evaluated quantitatively. In the reported methods, the shapes of GNSs were described by their length and number of branches, spherical core diameter, branch base width and overall size (Khoury and Vodinh, 2008; Senthil Kumar et al., 2008). Although they can describe roughness and morphology change of GNS to a certain degree during amalgamation, they are inconvenient and not accurate to show shape evolution of nanoparticles. The statistical results (Figure 1D) display that C value has a wide distribution in the absence of Hg^2+^. Upon increasing Hg^2+^ concentration, the distribution of C value becomes narrower. For example, the half-peak widths of the fitting Gauss curves in the absence and presence of 4 μM Hg^2+^ are 7.4 and 1.9, respectively. The C values for GNS at the Hg^2+^ concentration of 0, 4, and 8 μM are 18.22, 14.68, and 13.32, respectively. The results support Hg induced shape evolution of GNS. Based on the results, we propose a sensing route of our methodology as illustrated in Figure 2. When using GNS in the presence of AA, the concentration of Hg^2+^ can be easily monitored through the change of its absorption spectrum as a result of the morphology change of GNS due to formation of Au/Hg amalgams. When compared to the TSPR band, the LSPR is more susceptible to the shape evolution of GNSs induced by Hg^2+^.

### Optimization of experimental conditions for Hg^2+^ sensing

**Figure 3:**
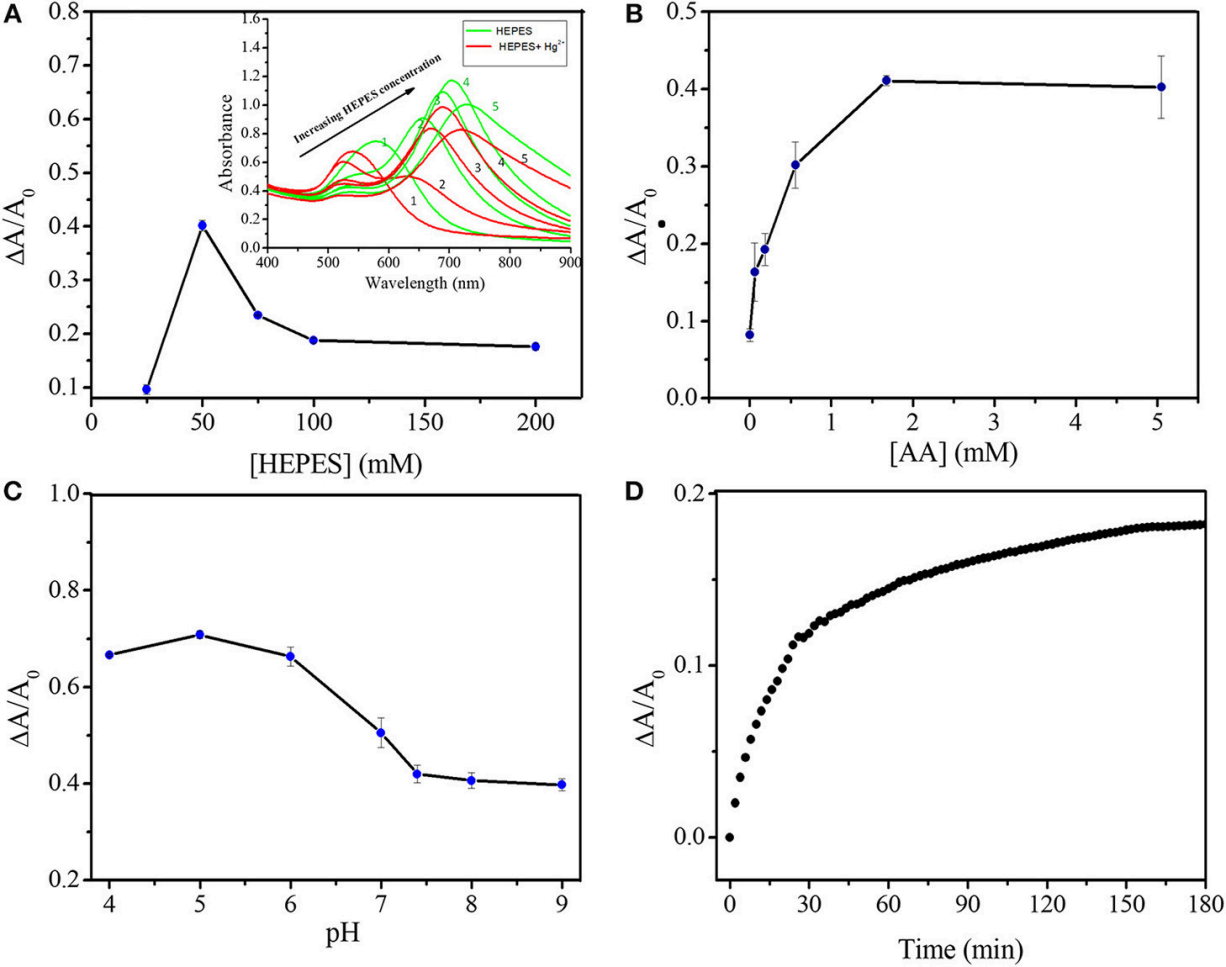
Optimization of experimental conditions on Hg^2+^ sensing. **(A)** ΔA/A_0_ of GNS solutions (pH 7.35) in the presence of 2 mM AA and 4 μM Hg^2+^ with incubation time of 102 min. Inset to A is the corresponding UV-Vis spectra of GNS solutions before (in red lines) and after (in blue lines) the addition of mercuric ions. The LSPR of GNS varies by increasing HEPES concentration (1–5 are corresponding to HEPES concentrations of 25, 50, 75, 100, and 200 mM, respectively). **(B)** Effect of AA concentration on ΔA/A_0_ of GNS solutions (pH 7.35) containing 4 μM Hg^2+^. **(C)** Effect of solution pH on the ΔA/A_0_ variation in the presence of 1.68 mM AA and 4 μM Hg^2+^. **(D)** Effect of reaction time on ΔA/A_0_ of GNS solutions (pH 5.0) in the presence of 1.68 mM AA and 1 μM Hg^2+^. GNS used in B-D were prepared in the presence of 50 mM HEPES.

To achieve high sensitivity, the LSPR of GNS, AA concentration, solution pH and the incubation time were systematically tested as shown in Figure 3. To minimize the effects of concentration and structure variation of GNS on quantitation, ΔA/A_0_, not ΔA, was applied to evaluate the spectra changes under various conditions in this study, where A_0_, is the maximum absorbance at the LSPR of GNS in the absence of Hg^2+^ and ΔA is the absorbance difference at the LSPR after addition of Hg^2+^. Firstly, GNSs with various structures were prepared in the presence of different concentrations of HEPES (Figure 3A) (Xie et al., 2007; Dam et al., 2012; Liu et al., 2014). By increasing HEPES concentration from 25 to 200 mM meanwhile keeping other parameters constant, GNSs with various LSPR wavelengths (580–730 nm) were prepared. The absorbance values at their corresponding LSPR wavelength increase upon increasing HEPES concentration and reach a maximum at 100 mM, with absorbance of 0.95 at 704 nm. Similar trend has also been reproted (de Puig et al., 2015; Chandra et al., 2016), where the role of HEPES was to reduce Au^3+^ and direct GNS growth, and the increase of HEPES concentration in synthesis procedure could result in longer branches. When the HEPES concentration is lower than 25 mM, no GNS was formed, with a support of no TSPR band. When using the GNS with an LSPR band at 656 nm, 4 μM Hg^2+^ induces a maximum ΔA/A_0_ change. A smaller change in ΔA/A_0_ was obtained when using GNSs with the LSPR wavelength longer than 656 nm, because they usually have longer branches (de Puig et al., 2015) that need greater amount of mercury to induce their structural change to form a spherical shape. HEPES itself has no apparent impact on Hg^2+^ determination, because GNS treated with centrifugation or adding more HEPES have almost the same response. The approximate concentration of GNS in testing solution is 0.23 nM, calculated by the total amount of HAuCl_4_ and the size of GNS obtained under TEM images. Secondly, the AA concentrations in the range of 0–5 mM were investigated, while keeping other conditions constant. As Figure 3B illustrates, increasing AA concentration leads to greater Hg^2+^ induced changes in the LSPR absorbance. When the concentration of AA is higher than 1.68 mM, the LSPR did not change further, mainly because Hg^2+^ ions were reduced completely by AA. Thirdly, the solution pH values from 4.0 to 9.0 were tested for Hg^2+^ sensing (Figure 3C and Figure S1), revealing that pH 5.0 is optimal. At pH < 5.0, GNS becomes unstable, while at higher pH values, formation of mercuric hydroxides is problematic. Finally, reaction time of 150 min was selected as shown in Figure 3D and Figure S2. Based on the above results, the optimal analytical conditions for quantitation of Hg^2+^ are GNS prepared in the presence of 50 mM HEPES, 1.68 mM AA, pH 5.0 and reaction time of 150 min.

### Sensitivity and selectivity

**Figure 4:**
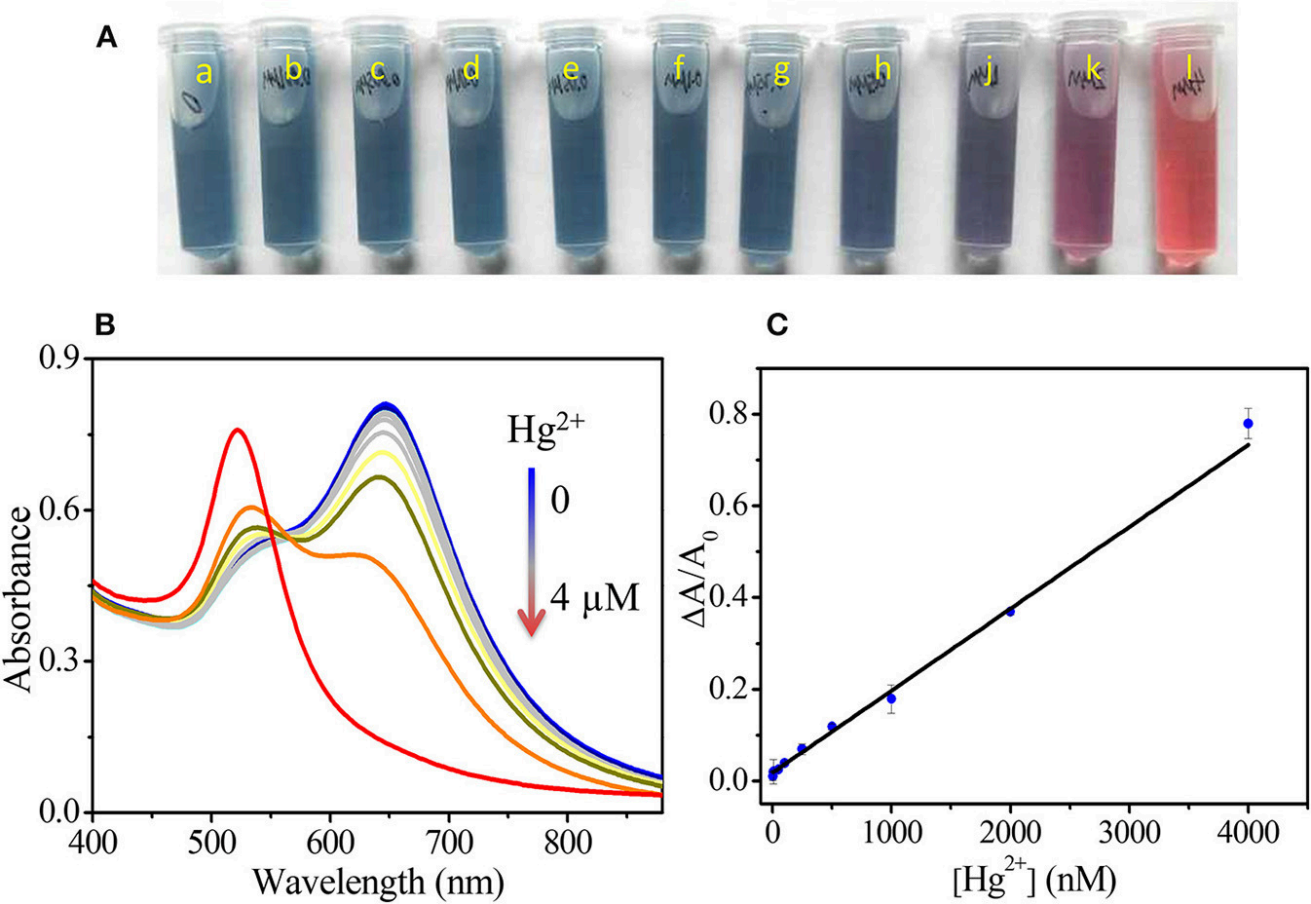
Photos **(A)** and UV-Vis spectra **(B)** of GNS solutions after addition of different amounts of Hg^2+^. a–j in A are corresponding to 0, 1, 5, 10, 50, 100, 250, 500, 1,000, 2,000 and 4,000 nM Hg^2+^, respectively. **(C)** A plot of ΔA/A_0_ vs. Hg^2+^ concentration. The fitting linear equation is ΔA/A_0_ = 0.01858 C_Hg_^2+^ + 0.00018.

To evaluate the sensitivity of our assay, the UV-Vis spectra of GNS solutions after the addition of different concentrations (1–4,000 nM) of Hg^2+^ solutions were taken. As Figure 4 shows, under the optimal conditions, increasing Hg^2+^ concentration (0, 1, 5, 10, 50, 100, 250, 500, 1,000, 2,000, and 4,000 nM) results in decreasing the LSPR absorbance and inducing greater blue shift of the LSPR. The solution color gradually changed from greenish-blue to red, due to the transformation of GNSs to spherical nanoparticles. The color change can be easily observed by the naked eye when Hg^2+^ concentration is above 250 nM. A linear relationship between ΔA/A_0_ and Hg^2+^ concentration was established (ΔA/A_0_ = 0.01858 C_Hg_^2+^ + 0.00018) with a coefficient of 0.997. The detection upper limit of our assay is 4,000 nM and the limit of detection (LOD) is 0.24 nM calculated by 3σ, which is lower than the values set by both EPA and WHO standards. When compared to the probe using gold nanorods, our assay is more sensitive for quantitation of Hg^2+^ as shown in Figure S3. We note that the sides of gold nanorod are capped with CTAB molecules, limiting the deposition of Hg (Hg^2+^) onto most of their surfaces. Some Hg (Hg^2+^) can only be deposited onto their two ends, leading to a slightly higher aspect ratio and insignificant changes in their LSTR absorbance. For example, addition of Hg^2+^ at 10,000 nM to gold nanorods solution under the same conditions only leads to a very small change in the LSPR absorbance (0.13) at 653 nm. When compared to other shaped nanomaterials toward Hg^2+^, GNS provides higher sensitivity due to its greater surface area. More importantly, the LSPR absorbance of GNS is highly sensitive to the change in its morphology. As a result, our method over most of the existing colorimetric methods (Lee et al., 2007; Li et al., 2008; Xue et al., 2008; Chai et al., 2010; Du et al., 2011) is more sensitive for Hg^2+^ quantitation, also see Table S1. Unlike most reported nanoparticle based approaches that are dependent on aggregation or disaggregation, our assay is based on Hg^2+^ induced morphological transformation of GNS. In addition, our assay is more sensitive than atomic absorption spectroscopy (Hatch and Ott, 1968) and fluorescence methods (Chen et al., 2015).

**Figure 5:**
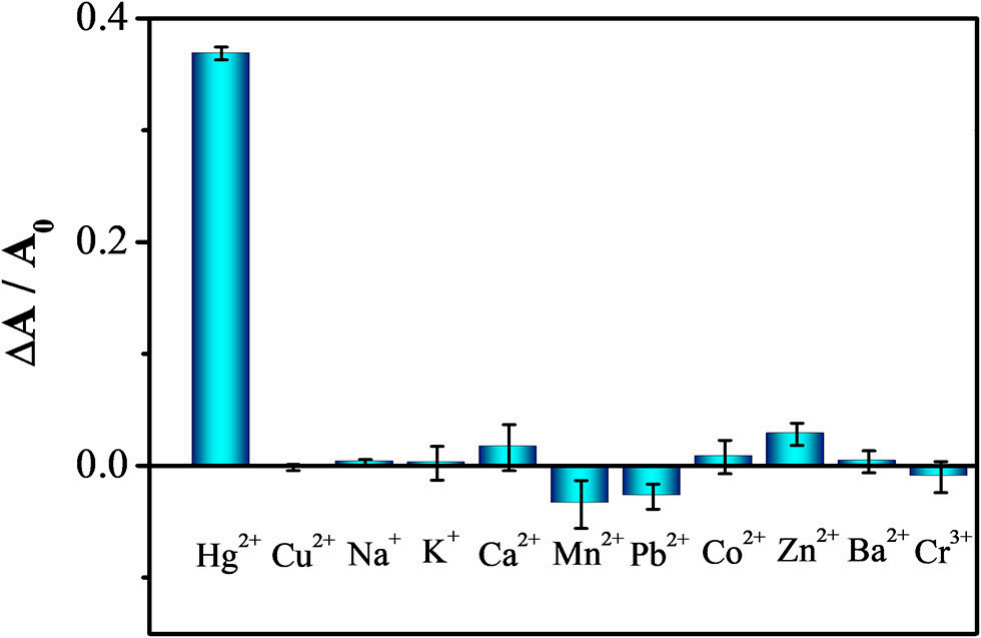
Selectivity of GNS toward 2 μM Hg^2+^ over 100 μ M of other metal ions, including Cu^2+^, Na^+^, K^+^, Ca^2+^, Mn^2+^, Pb^2+^, Co^2+^, Zn^2+^, Ba^2+^ and Cr^3+^.

Our assay also has excellent selectivity toward Hg^2+^ (2 μM) over the tested metal ions (100 μM), including Cu^2+^, Na^+^, K^+^, Ca^2+^, Mn^2+^, Pb^2+^, Co^2+^, Zn^2+^, Ba^2+^, and Cr^3+^, as shown in Figure 5. The ability of metal to form alloys with mercury is related to its solubility in mercury. In general, metals with similar property and near in the Periodic Table have higher solubility (Gumiński, 2015). Thus, when compared to other metals, Au forms alloys with Hg more strongly. In addition, AA cannot reduce most of the tested metal ions at pH 5.0, but can form complexes with some of them to further minimize their interactions with the GNSs (Martell, 1982). The role of AA in stabilizing the GNS to minimize the salt inducing aggregation of GNSs is also evident.

### Mercury quantitation in real samples

Having such a high sensitivity, selectivity, and stability in high-ionic-strength solution, our assay is expected to be suitable for Hg^2+^ quantitation in real samples. To validate our assay for real applications, Hg^2+^ spiked tap, pond, and river water samples were analyzed, with results listed in Table 1. The Hg^2+^ concentrations in the three samples (each with three measurements) were found to be 0.29 (±0.03), 0.62 (±0.03) and 0.71 (±0.06) nM, respectively. Their concentrations are all below the allowable standards of EPA and WHO. The recoveries of Hg^2+^ in the spiked samples are satisfactory, showing the potential of this assay for Hg^2+^ quantitation in real samples.

**Table 1:** Quantitationof Hg^2+^ in water samples (*n* = 3).

**Sample**	**Added**	**Detected**	**Recovery**	**RSD**
	**(nM)**	**(nM)**	**(%)**	**(%)**
Tap water	0	0.29	/	9.76
	200	203.3	102.5	0.83
Pond water	0	0.62	/	3.71
	300	311.4	103.6	0.62
River water	0	0.71	/	8.14
	1,000	1071.5	107.2	0.3

## Conclusion

A highly sensitive methodology for mercury quantitation using GNS probe was proposed. The assay is based on the formation of Au-Hg amalgamates on GNSs in the presence of AA, leading to significant changes in their LSPR absorbance. With very high sensitivity of the LSPR absorbance of GNS to its morphology changes and specificity of GNS toward Hg, our assay is highly sensitive (LOD 0.24 nM) and selective for Hg^2+^ quantitation. In this study, we also found that AA plays several important roles, acting as a reducing agent to reduce Hg^2+^, a capping agent to stabilize the GNS, and a complexing agent to form complexes with the potential interfering species. Using the average circular ratio of GNSs, the Hg induced morphological transformation of GNSs can be described easily. Based on the fact that the assay is sensitive and selective for Hg^2+^ quantitation, we can foresee its application for quantitation of toxic organic mercury species.

## Author contributions

DX and QL designed the work and were part of the manuscript write-up. DX, SY, YY, and SW carried out the experiments, interpreted some of the results and were also involved in the manuscript preparation. ZY revised and edited the manuscript. All the authors reviewed the manuscript and have agreed to its publication.

### Conflict of interest statement

The authors declare that the research was conducted in the absence of any commercial or financial relationships that could be construed as a potential conflict of interest.
