# Supplementary material for: Sensitive Colorimetric Hg2+ Detection via Amalgamation-Mediated Shape Transition of Gold Nanostars
Source: Front Chem. 2018 Nov 27;6:566. doi: 10.3389/fchem.2018.00566 (PMC6277514; doi:10.3389/fchem.2018.00566)
Supplement: Supplementary file 1 [file Data_Sheet_1.pdf]

Supplementary Material:

Sensitive colorimetric Hg^{2+} detection via amalgamation-mediated shape transition of gold nanostars

Dong Xu[1,2], Shufang Yu[1,2], Yueqin Yin[1,2], Suyang Wang[1,2], Qinlu Lin[1,2*] and Zhiqin Yuan[3]

1. *National Engineering Laboratory for Rice and By-products Further Processing, College of Food Science and Engineering, Central South University of Forestry and Technology, Changsha, Hunan, China,*
2. *Hunan Key Laboratory of Processed Food for Special Medical Purpose, College of Food Science and Engineering, Central South University of Forestry and Technology, Changsha, Hunan,China*
3. *State Key Laboratory of Chemical Resource Engineering, Beijing University of Chemical Technology, Beijing, China*

**Corresponding author.*

E-mail adress: LinQL0403@163.com, Tel/Fax: +86-731-8562-3240

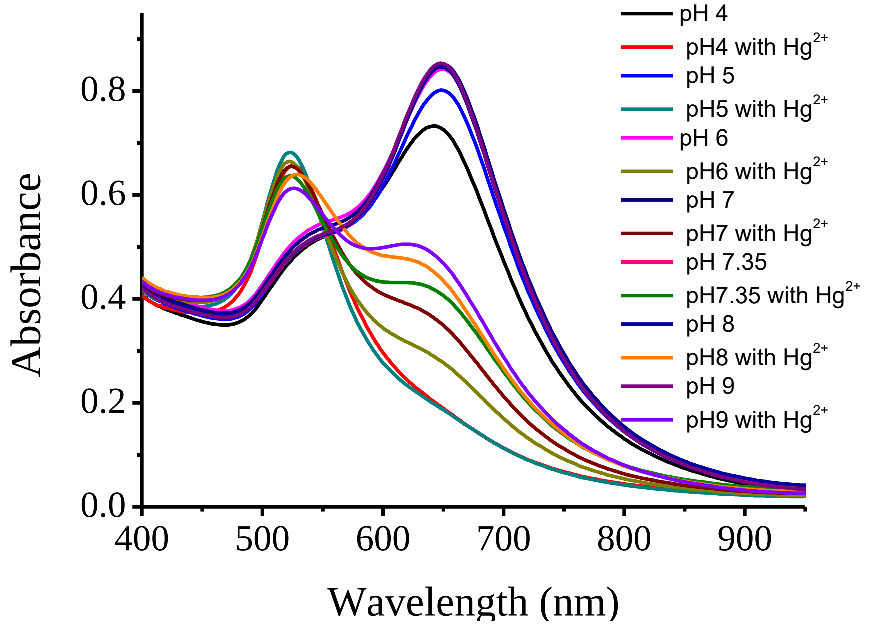

Figure S1.UV-Vis spectra of GNS solutions in the presence of 1.68 mM AA with and without addition of 4 μM Hg^{2+} at different pH values for 102 min.

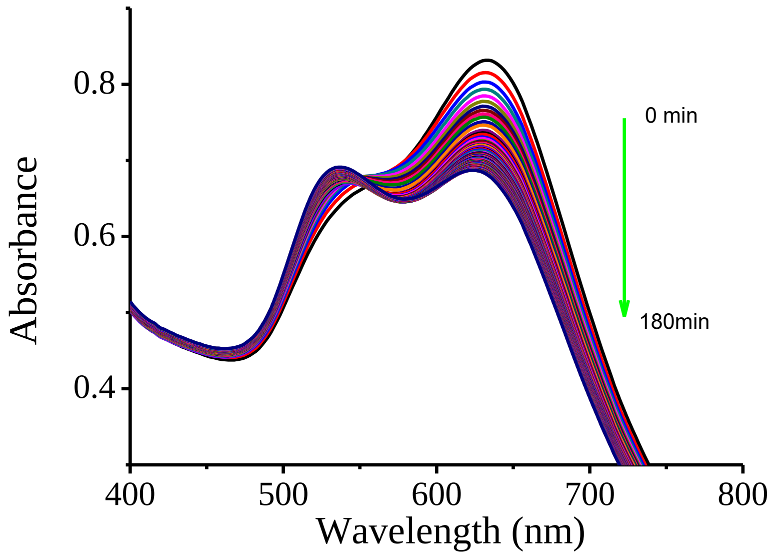

Figure S2. Time evolution of UV-Vis spectra of GNS solution containing 1.0 μM Hg^{2+} with time interval of 2 min. Other conditions are the same as in Fig 3.

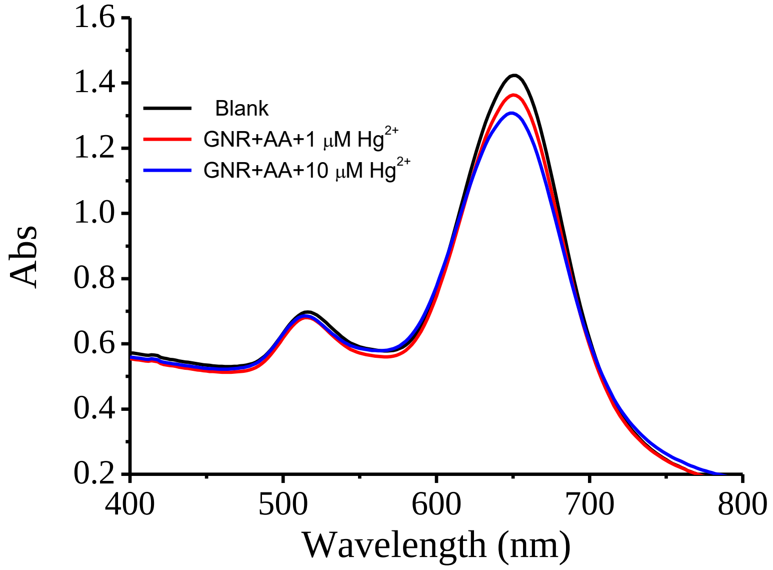

Figure S3. UV-Vis spectra of gold nanorod solutions containing 1.68 mM AA at pH 5.0 after the addition of 0, 1 and 10 μM Hg^{2+} with incubation time of 150 min.

Table S1. The comparision of this work with some established gold nanomaterial-based colorimetric Hg^{2+} assays

Materials	Method	Detection range	Detection limit	Ref
Trithiocyanuric acid functionalized gold nanoparticle	Aggregation	5×10^{-9} - 1×10^{-6} M	2.8 nM	1
Citrate-capped gold nanoparticle	Aggregation	1-60 nM	2.9 nM	2
ssDNA-gold nanoparticle	Aggregation	25-750 nM	50 nM	3
Gold nanorod coated with 6-mercaptopurine	Anti-aggregation	1–100 nM	0.48 nM	4
Gold nanoparticle	Anti-aggregation	0-680 nM	11.9 nM	5
Gold nanoparticle	Aggregation and growth	8.76 nM – 127μM	8.76 nM	6
Aptamer-gold nanoparticle	Growth	0-80 nM	3 nM	7
DNA-gold nanoparticle	Aggregation	5-50 nM	1.5 nM	8

1. Wang, J., Fang, X., Cui, X., Zhang, Y., Zhao, H., Li, X., and He, Y. (2018). A highly sensitive colorimetric probe for Cd^{2+}, Hg^{2+} and ascorbic acid determination based on trithiocyanuric acid-AuNPs. *Talanta* 188**,** 266-272. doi:10.1016/j.talanta.2018.05.084
2. Sener, G., Uzun, L., and Denizli, A. (2014). Lysine-Promoted Colorimetric Response of Gold Nanoparticles: A Simple Assay for Ultrasensitive Mercury(II) Detection. *Anal. Chem.* 86**,** 514-520. doi:10.1021/ac403447a
3. Chen, G.-H., Chen, W.-Y., Yen, Y.-C., Wang, C.-W., Chang, H.-T., and Chen, C.-F. (2014). Detection of Mercury(II) Ions Using Colorimetric Gold Nanoparticles on Paper-Based Analytical Devices. *Anal. Chem.* 86**,** 6843-6849. doi:10.1021/ac5008688
4. Bi, N., Hu, M.H., Xu, J., and Jia, L. (2017). Colorimetric determination of mercury(II) based on the inhibition of the aggregation of gold nanorods coated with 6-mercaptopurine. *Microchimica Acta* 184**,** 3961-3967. doi:10.1007/s00604-017-2427-5
5. Sun, X., Liu, R., Liu, Q., Fei, Q., Feng, G., Shan, H., and Huan, Y. (2018). Colorimetric sensing of mercury (II) ion based on anti-aggregation of gold nanoparticles in the presence of hexadecyl trimethyl ammonium bromide. *Sensor. Actuat. B-Chem.* 260**,** 998-1003. doi:10.1016/j.snb.2018.01.083
6. Liu, Y., Liu, Y.L., Xu, L., Li, J., Liu, X.Y., Liu, J.S., and Li, G.Y. (2017). Highly selective, colorimetric detection of Hg^{2+} based on three color changes of AuNPs solution from red through sandy beige to celandine green. *Sensor. Actuat. B-Chem.* 249**,** 331-338. doi:10.1016/j.snb.2017.04.116
7. Tan, L., Chen, Z., Zhang, C., Wei, X., Lou, T., and Zhao, Y. (2017). Colorimetric Detection of Hg(2+) Based on the Growth of Aptamer-Coated AuNPs: The Effect of Prolonging Aptamer Strands. *Small.* 13(14), 1603370. doi: 10.1002/smll.201603370
8. Peng, C.F., Pan, N., Xie, Z.J., and Wu, L.L. (2016). Highly sensitive and selective colorimetric detection of Hg^{2+} based on the separation of Hg^{2+} and formation of catalytic DNA-gold nanoparticles. *Anal. Methods* 8**,** 1021-1025. doi:10.1039/c5ay02843d
